# Changing climates, compounding challenges: a participatory study on how disasters affect the sexual and reproductive health and rights of young people in Fiji

**DOI:** 10.1136/bmjgh-2023-013299

**Published:** 2023-12-15

**Authors:** Nabreesa Murphy, Tamani Rarama, Alanieta Atama, Ilaisa Kauyaca, Kelera Batibasaga, Peter Azzopardi, Kathryn J Bowen, Meghan A Bohren

**Affiliations:** 1 Gender and Women's Health Unit, Nossal Institute for Global Health, The University of Melbourne School of Population and Global Health, Melbourne, Victoria, Australia; 2 Fiji Youth Sexual and Reproductive Health and Rights Alliance (FYSA), Nadi, Fiji; 3 Reproductive and Family Health Association of Fiji (RFHAF), Nadi, Fiji; 4 Centre for Adolescent Health, Murdoch Children's Research Institute, Department of Paediatrics, University of Melbourne, Melbourne, Victoria, Australia; 5 Adolescent Health and Wellbeing Program, Telethon Kids Institute, Adelaide, South Australia, Australia; 6 Melbourne Climate Futures, Melbourne School of Population and Global Health, University of Melbourne, Melbourne, Victoria, Australia; 7 Research Institute for Sustainability—Helmholtz Centre Potsdam, Potsdam, Brandenburg, Germany

**Keywords:** Health policies and all other topics, Prevention strategies, Public Health, Qualitative study

## Abstract

Pacific youth are at the forefront of the climate crisis, which has important implications for their health and rights. Youth in Fiji currently bear a disproportionate burden of poor experiences and outcomes related to their sexual and reproductive health and rights (SRHR). There is limited information about how the increasing climate impacts may affect their SRHR, and what the implications may be for climate action and disaster risk reduction. We aimed to explore the experiences of 21 Fijian youth in fulfilling their SRHR when living through multiple natural hazards. We conducted 2 workshops and 18 individual semistructured interviews using visual and storytelling methods. Irrespective of the type of hazard or context of disasters, participants identified limited agency as the main challenge that increased SRHR risks. Through reflexive thematic analysis, we identified four themes centred around ‘youth SRHR agency’; (1) information and knowledge, (2) community and belonging, (3) needs and resources, and (4) collective risks. These themes encompassed multiple factors that limited youth agency and increased their SRHR risks. Participants highlighted how existing challenges to their SRHR, such as access to SRHR information being controlled by community gatekeepers, and discrimination of sexual and gender diverse youth, were exacerbated in disasters. In disaster contexts, immediate priorities such as water, food and financial insecurity increased risks of transactional early marriage and transactional sex to access these resources. Daily SRHR risks related to normalisation of sexual and gender-based violence and taboos limited youth agency and influenced their perceptions of disasters and SRHR risks. Findings offer important insights into factors that limited youth SRHR agency before, during and after disasters. We underscore the urgency for addressing existing social and health inequities in climate and disaster governance. We highlight four key implications for reducing youth SRHR risks through whole-of-society approaches at multiple (sociocultural, institutional, governance) levels.

**WHAT IS ALREADY KNOWN ON THIS TOPIC**

- The impacts of climate change disproportionately affect the health and rights of those who are least responsible for the climate crisis. Youth make up 44% of the population in Fiji, and currently experience many challenges to realisation of their sexual and reproductive health and rights (SRHR). There is limited information about the effects of increasing climate impacts on their SRHR. As SRHR is linked to health equity and sustainable development, understanding youth experiences is key to reducing SRHR risks in future.

**WHAT THIS STUDY ADDS**

- This study contributes valuable insights from Fijian youth about how they experience risks to their SRHR in the context of increasing climate-related disasters. Using visual and story-telling methods enabled us to ground the research in Fijian contexts and prioritise the voices and worldviews of Fijian youth. We report several factors that affected youth SRHR agency and increased their SRHR risks before, during, and after disasters. Findings highlight how climate impacts exacerbate existing challenges and inequities.

**HOW THIS STUDY MIGHT AFFECT RESEARCH, PRACTICE OR POLICY**

- There are four key implications from our research to increase youth SRHR agency and reduce future risks. We advocate for formalising comprehensive sexuality education in school curriculums to address the identified need for accurate SRHR information and knowledge. We underscore the importance of taking a whole-of-society approach to increase community awareness and understanding of SRHR risks. We note the creative and vital contributions by youth and advocate for their meaningful inclusion in climate and disaster risk governance. Finally, we highlight how the intersections of food, water, and financial insecurities increased SRHR risks, and emphasise the importance of inter-sectoral collaboration to strengthen youth SRHR agency.

## Introduction

The climate crisis is a significant and mounting threat to human and ecological health.^1 2^ Pacific Island nations have advocated for the recognition of climate impacts as issues of equity and justice, noting that countries and populations who are least responsible for the crisis are bearing the brunt of the impacts.^3 4^ Despite having limited responsibility for climate change, the Pacific is simultaneously experiencing cascading effects of multiple slow-onset hazards (such as coastal inundation and saltwater intrusion into freshwater sources) and sudden-onset hazards (such as cyclones and floods).^4^ Climate impacts result in worsening food insecurity, water scarcity and economic insecurity, and threaten the health, lives and livelihoods of Pacific communities. Globally, youth are among the most at risk from climate impacts, both from direct impacts on their mental and physical health, and from indirect impacts related to worsening food, water and economic insecurities. However, due to institutional and structural barriers, youth have limited access to decision-making processes that shape climate action.^5–7^ In 2021, 18% of the population in the Pacific Region were youth (aged 15–24 years), and these youth are at the forefront of the climate crisis.^8^ Pacific youth are instrumental in highlighting the inequitable impacts of the climate crisis, as demonstrated by a landmark Pacific youth-led campaign that successfully took the issue of climate justice to the International Court of Justice in 2023.^9^

Climate and disaster risks are the consequences of hazards interacting with social and political conditions that increase exposure and vulnerability of people and places.^10^ The social and political creation of risks reinforces climate impacts as justice issues, disproportionately affecting people already experiencing structural and systemic inequities, including limited access to information, health services and financial resources.^11 12^ Addressing these underlying climate impact drivers is vital for both disaster risk reduction and climate change adaptation.^13^

The increasing frequency of global climate-related disasters has highlighted that siloed approaches for analysing and governing disaster risk are no longer adequate. Consequently, the United Nations Office for Disaster Risk Reduction (UNDRR) recommends transdisciplinary approaches to understand the systemic nature of risks within evolving social and environmental contexts.^14^ Importantly, UNDRR underscores the salience of context—such as local social values and belief systems—in understanding drivers of systemic risk.^14^ Highlighting the synergies in global priorities, community engagement and inclusive approaches to sustainable development are also recommended in both the Sendai Framework for Disaster Risk Reduction, and the 2030 Sustainable Development Goals.^15 16^

### Considering the climate impacts on Fijian youth sexual and reproductive health and rights

As a country with high exposure to tropical cyclones, rising sea levels, floods and landslides, Fiji is one of the world’s most vulnerable countries to climate impacts.^17^ Community-engaged and inclusive approaches to risk reduction are particularly important for Fijian youth, as their ability to contribute in their communities is based on socially prescribed roles aligned with hierarchies related to factors such as lineage, age and gender.^18^ Approximately 45% of the Fijian population are under 25 years old, and youth aged 15–24 represent almost 17% of the national population.^8 19^ In Fiji, definitions of youth vary, ranging between 15 and 35 years, as cultural factors linked to marriage, employment or positions of authority determine who is considered an adult.^18 20^ Climate modelling estimates that 30% of the population of Fiji are at risk from multiple compounding hazards.^21^ Given the large youth population in Fiji, the increasing climate impacts have substantial implications for Fijian youth. While the UN definition of youth is 15–24, for practical and ethical reasons to respect participant confidentiality and autonomy, we adopted an operational definition of youth as 18–26 years.

Sexual and reproductive health and rights (SRHR) emphasises that fulfilment of sexual and reproductive health relies on the ability to realise sexual and reproductive rights, and the freedom to do so without coercion, discrimination, violence and stigma.^22^ SRHR are essential indicators of youth health and well-being throughout the life course, with intergenerational impacts.^23^ Inequities, such as those related to poverty and gender, shape all aspects of youth health and well-being.^23^ For instance, the ability of youth to meet contraceptive needs and delay early pregnancy or marriage depends on access to resources (such as finances), opportunities (such as education and skills-based training) and services (such as SRHR services). Consequently, the extent to which youth SRHR is fostered or compromised affects their health and social capabilities across their lifetime, and influences a healthy start of life for the next generation. Compared with the general population, Fijian youth experience a disproportionate burden of negative SRHR experiences and outcomes, which are strongly influenced by social factors including taboos, stigma and societal expectations.^24–26^ High rates of adolescent pregnancy and birth are coupled with low contraceptive prevalence rates, with 81% of 15–19 year old and 68% of 20–24 year old married women not using any contraceptive method.^19^ Less than half (39.8%) of men and (28.7%) of women aged 15–24 reported using condoms with sexual partners in the last 12 months, and the met need for family planning in women aged 15–49 remains low at only 10%.^19^ Over half of Fijian women 15 years and older have experienced sexual or gender-based violence at least once in their lifetime, which is well above the global rate of 26%.^27^ Persistent gender inequities, coupled with patriarchal beliefs and practices are likely to be contributing factors to the poor SRHR outcomes and experiences. For example, 15.9% of Fijian women aged 15–19 years have a spouse 10 or more years older than them.^19^ Furthermore, intersections between multiple social identities (such as gender, sexual orientation and socioeconomic status) influence differential experiences of power and marginalisation among youth, increasing challenges to fulfilling their SRHR.^28^

There is limited knowledge about how Fijian youth navigate their SRHR in the context of increasing climate impacts. Humanitarian practitioners emphasise youth as a diverse population with different SRHR needs to adults, who also experience differential impacts based on social identities that intersect with age, such as sexual and gender diversity or disability.^29^ Pacific grassroots organisations note increased rates of sexual and gender-based violence towards people with sexual and gender diversity before and after disasters.^28 30^ Consequently, they advocate for mainstreaming sexual and gender diversity in disaster risk reduction policies and practices.^30^ Supporting this, Murphy and colleagues’ recent scoping review of humanitarian responses to the 2020 Tropical Cyclone Harold in Fiji, Vanuatu and Tonga also highlighted how a binary perspective of gender increased risks of invisibility and exclusion for youth with sexual and gender diversity.^31^

The priorities and needs of youth in all their diversity are currently under-represented in SRHR research and programmes in humanitarian settings.^32^ In the aftermath of the 2020 Tropical Cyclone Harold, Pacific community organisations and SRHR service providers demonstrated their key role in meeting some of the SRHR needs of youth, through trusted partnerships and community networks.^33^ As Pacific youth experience multiple challenges to realisation of SRHR, their perspectives are crucial to understand their risks related to climate impacts. Additionally, SRHR is an essential indicator of gender equality and interlinked with sustainable development. The ability of youth to fulfil their SRHR is therefore critical to ensuring civic participation and fostering community resilience for sustainable development. The aim of this paper is to explore how Fijian youth fulfil their SRHR in the multihazard contexts they live in, with a specific focus on climate impacts.

## Methods

### Patient and public involvement

We conducted a participatory qualitative study, based on a collaboration between the Fiji Youth Sexual and Reproductive Health and Rights Alliance (FYSA) and the University of Melbourne, Australia. All aspects of the study from development of research questions, research methods, analysis, write-up and dissemination were conducted through this collaboration. In addition to research outputs, dissemination materials (reports, infographics) are being disseminated to youth participants, community members and key stakeholders at the community, country and regional levels. Further details about our collaboration are included in online supplemental appendix A.

10.1136/bmjgh-2023-013299.supp1 Supplementary data

### Theoretical approach

Guided by Freire’s theory of praxis^34^ and the Indigenous Fijian Vanua Research Framework,^35^ both of which prioritise transformative research for action, we used qualitative and participatory approaches rooted in mutual respect, reflection and collaboration. Guided by our shared vision for advancing youth SRHR, we aimed to identify opportunities for transformative action to reduce climate impacts on youth SRHR. We viewed youth as a diverse group with intersecting identities and experiences that may influence their perceptions of SRHR and climate risks. This included deliberately looking beyond the binary definitions of gender to understand how gender and sexual diversity influence climate and disaster risks. We intended to gain an intersectional understanding of youth SRHR experiences and identify multiple opportunities for advancing social justice. Our reporting is guided by the standards for reporting qualitative research (online supplemental appendix B).^36^

10.1136/bmjgh-2023-013299.supp2 Supplementary data

### Study setting

This study took place in Ba, Lautoka and Nadi, in the Western district of Fiji. FYSA identified the Western district as an area with low youth engagement and high youth SRHR needs. The West was also the epicentre of the COVID-19 outbreak in Fiji in 2020 and 2021 and and was under lockdown during multiple cyclones and floods, increasing multiple social vulnerabilities.^37^

### Participants and recruitment

Participants were eligible for inclusion if they were living in Lautoka, Ba, or Nadi, had experienced disasters, were able to participate in interviews in English or i-Taukei Fijian. We recruited participants by distributing a flyer on social media, emailing colleagues at Pacific organisations, through professional networks of the research team, and snowball sampling. As 18 was the legal voting age, only participants who were 18 years and over were eligible to participate. Our target population was youth aged 18–24. However, one participant who attended the first workshop had just turned 26. Acknowledging the fluidity of youth definitions in the Pacific, and to respect participant autonomy we chose to include the 26 year old in the study.

### Data collection and management

A total of 21 youth participants participated in our study. NM and TR facilitated two in-person workshops and conducted 18 qualitative interviews between August and October 2022, supported by two youth facilitators (AA and IK) and an SRHR and mental health counsellor (KB). In workshop 1, held over 1 day with 21 participants, we used three activities to encourage participants to use photographs and drawings to consider factors that affect youth SRHR in disaster contexts. Using visual and storytelling methods is aligned with the principles of the Fijian Vanua Research Framework, as it respects Fijian cultural contexts and relational ways of knowledge exchange.^35^ At the end of workshop 1, we posed three questions to participants: (*1) how do youth keep their health, bodies, and rights safe after disasters? (2) How did youth help each other keep health, bodies, and rights safe? (3) How can youth SRHR be improved in future disasters?* Participants had the option of answering the questions using their preferred visual or narrative methods. Once they answered the questions, participants contacted NM for an individual interview to elaborate on their answers. NM and TR conducted 18 open-ended, participant-guided qualitative interviews, 15 in English and three in Vosa Vakaviti (Indigenous Fijian language). Participants used a mix of photographs, illustrations and written narratives to answer the three questions. We used questions such as ‘*what would you like to tell us about this photo/answer/drawing’* to elicit further information during interviews.

In workshop 2, a full-day attended by 12 participants, we shared findings from an initial rapid analysis of the individual interviews. This was to give participants the opportunity to provide feedback, reflect on, add to and discuss findings. Following the discussions, participants created stories, posters and illustrations to highlight key findings that they found most important or wanted to focus on for action beyond the project. This helped us inform our plans for advocacy and knowledge translation. Both workshops were held in Lautoka, all meals were provided, and participants were reimbursed for their travel (FJD 20 for those travelling from Ba and Nadi, and FJD 10 for those in Lautoka). We reimbursed the 18 individual interview participants (FJD 30) for their time, travel to interview sites, and mobile data use.

Data generated from the project included audio-recordings and written transcripts from 2 full days (16 hours) of workshops, 17 hours of individual interviews with 18 youth participants. Additional data from individual interviews included narratives combined with 17 photographs and 12 illustrations. Visual data generated by participants from the two workshops included narratives with nine photographs and nine poster illustrations from workshop 1 and four illustrations and two stories from Workshop 2. In addition, the research team created four posters of the identified themes during the data analysis, which participants added to during workshop 2 as part of participatory coproduction of knowledge. The workshop discussions and interviews conducted in English were audio-recorded and transcribed verbatim by NM. The interviews in Vosa Vakaviti were audio-recorded in Vosa Vakaviti and the deidentified recording was transcribed in English by IK. We stored all participant-derived illustrations, photographs and narratives securely in a password-protected OneDrive account.

### Data analysis

We used reflexive thematic analysis as a team to analyse the data.^38^ Reflexive thematic analysis views knowledge as situated within sociocultural and political contexts and shaped by the research processes, which aligned with our theoretical approach. It facilitated a collaborative and participatory approach to knowledge production. Four members of the research team (NM, TR, AA and IK) individually analysed the interview recordings and transcripts before meeting in person to discuss as a group. Over 4 days, we collaborated as a team to discuss, analyse and generate all codes and themes. Reflexive thematic analysis views subjectivity as an essential resource to the analysis process.^38^ That is, who we are—our own personal identities and values and our disciplinary perspectives—shaped how we engaged with the research process and the analysis. Our discussions started with what stood out for each of us from reading the narratives and transcripts, reflecting our own positionalities, experiences and perspectives. Through discussion and reflection, we generated initial codes (patterns of meaning). We then engaged in a process of grouping similar codes into themes and further refining the codes and themes as our understanding of participant accounts and narratives evolved. An evolving coding process reflects good practice in reflexive thematic analysis, enabling us to fully engage with the data to generate nuanced, rich analytic insights.^38^ Staying close to participant accounts, our analysis generated a central concept (overarching theme) and four main themes structured around the central concept. The collaborative meaning-making process was aligned with our theoretical approaches, enabling us to share power and knowledge, and generate contextually relevant understandings of participant experiences.

### Ethics and researcher reflexivity

We obtained written informed consent from all participants, including separate and specific consent for use of all participant-derived photographs and illustrations. To minimise distress to participants, we developed a distress prevention, mitigation and response protocol to guide the research activities, and used rights-based approaches to encourage participants to focus on solutions or actions for positive change. KB attended both workshops and was available if participants required any counselling or referral to clinical SRHR services. To protect privacy and confidentiality of participants and their communities, we encouraged participants to use non-identifying methods such as images of scenery or objects, illustrations and narratives to discuss and respond to research questions. To maintain confidentiality, we used Fijian flower names as pseudonyms for all participants.

NM grew up in an island community and became aware of gender, health and climate inequities from an early age. She has professional experience of clinical emergency medicine, community public health and SRHR research and advocacy. KB is an SRHR and mental health counsellor, and TR, AA and IK have experience in grassroots advocacy and programme delivery on intersectional youth SRHR, climate change, disasters and mental health issues. Research supervisors (MAB, PA and KJB) have combined expertise in adolescent health, climate and health, SRHR and qualitative and participatory methods. The research team was driven by intersectional feminist perspectives, and our collective experiences guided how we conducted the research and interpreted our findings. As a group diverse in our ages, gender identities and sexualities, our combined personal and professional experiences helped us gain a contextual and nuanced understanding of participant experiences. See online supplemental appendix A for further reflections on our collaborative approach.

## Findings

Twenty-one youth participated in the study (table 1: sociodemographic characteristics). Participants were diverse in their ages, sexual orientation and gender identities (including young men, women and gender non-conforming youth), ethnicities (i-Taukei Fijian and Indo-Fijian) and primary residence. We chose not to report the individual gender identities and sexual orientations of participants to maintain confidentiality and minimise risks of being identified. While residing in the three study sites, participants also drew on their experiences of living in various rural and remote communities.

**Table 1:** Sociodemographic characteristics of participants

	Participants (n=21)
Primary residence	
Lautoka	15
Ba	3
Nadi	3
Age	
18–21	9
22–25	11
26–29	1
Ethnicity	
i-Taukei Fijian	19
Indo-Fijian	2

**Figure 1:**
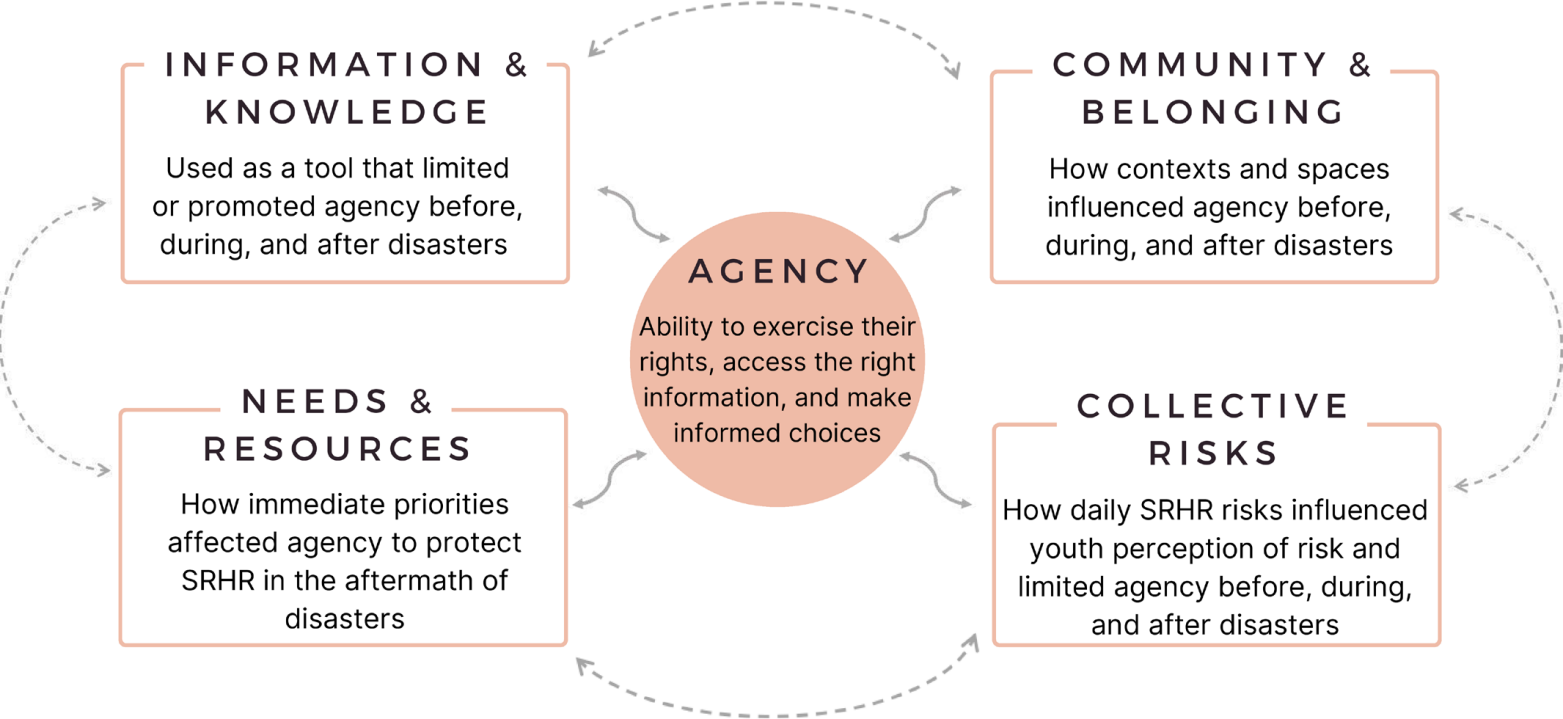
Summary of the four interconnected themes: (1) information and knowledge, (2) community and belonging, (3) needs and resources, and (4) collective risks, that influenced the overarching theme of agency before, during, and after disasters.

We found that irrespective of the types of hazards or the contexts of disasters, limited agency was the main challenge that increased youth SRHR risks before, during and after disasters. Participants described multiple factors that influenced agency and increased SRHR risks both in non-disaster settings and during disaster response and recovery. While all participants noted that existing gender inequity increased risks for young women, the gender and sexual diversity of the group highlighted shared structural factors that affected SRHR agency and increased risks for all youth. Through reflexive thematic analysis, we identified SRHR agency as a central concept, with four interconnected themes that influenced youth agency to protect their SRHR (figure 1). We defined agency as the ability to exercise their SRHR, access the right information and make informed choices. The four themes centred around agency were (1) information and knowledge, (2) community and belonging, (3) needs and resources and (4) collective risks.

Participants described factors that restricted agency and increased their SRHR risks in non-disaster settings, and factors that further challenged agency and compounded SRHR risks during and after disasters. We elaborate on each of the four main themes in the following sections, using participant-derived narratives, photographs and illustrations from the workshops and interviews.

### Information and knowledge as a tool that limited or promoted agency before, during and after disasters

Participants reported that limited individual and community knowledge about SRHR resulted in youth being unaware of their SRHR risks in non-disaster contexts, which further increased their risks in disasters. Community decision-makers and elders often acted as gatekeepers of knowledge and information. As explained by Rosi, elders controlled what information they allowed into the communities, and who could access it. Rosi stated that according to village elders, youth were too young for SRHR information and training, despite high rates of adolescent pregnancies in her village. Elders blamed the increase in adolescent pregnancies on education, saying that what youth learnt in school was leading to pregnancy. Consequently, community gatekeepers became even more selective about what kind of information they permitted to be shared within their villages, and what education youth were allowed to access.

Most [community outreach] trainings that come to the community, [elders] would ask… what’s the training about, [before] they would allow [or] cancel it…[elders] say you’re too young for that [SRHR] topics…[it’s] hard because in my community, young people [are] giving birth at age of 17, 18…[but] the elders have said this is what learn from school. They’re bringing it back from school. So they are more precise [selective] on what kind of training or education comes in the community, or what they learn in school – Rosi

At the same time, participants had inconsistent views about the usefulness of sexuality education (called family life education (FLE)), in schools in Fiji. Buasala expressed frustration that in her school the focus of FLE was on decorating their home. She questioned the purpose of FLE, as while they were learning how to decorate their homes, adolescent girls were getting pregnant and leaving school.

‘Family life [education in schools] wasn’t about family life but about how you want your home to look like…[meanwhile] there was always one girl getting pregnant…getting out of school. So we’re like: ‘what are we here for’? – Buasala

Draunividi had a different experience of FLE and described it as very useful and informative. She explained that sexuality education at school enabled her and her peers to understand and know how to discuss SRHR issues.

[FLE] was educational …It was related to SRHR, sex education and your body, your health, and your rights. So from an early stage to adolescence to adults, all that we have learned in FLE classes, it really motivated and educated our mind and [helped know how] to talk about it …it really brightened up our minds’ – Draunividi

Participants reported that the inconsistent and limited access to SRHR information pushed Fijian youth to seek information from unreliable and unsafe sources. Buasala explained that youth were often using internet pornography as a source of information about sex. She therefore highlighted the need for accurate and accessible SRHR information.

‘Fiji was rated as one of the top countries for Google searches for porn…that just shows young Fijians are very curious about sex, but no one is teaching them so they are going to these platforms …It’s not a good place to learn about sex…We really want to know but no one is talking about it’ – Buasala

**Figure 2:**
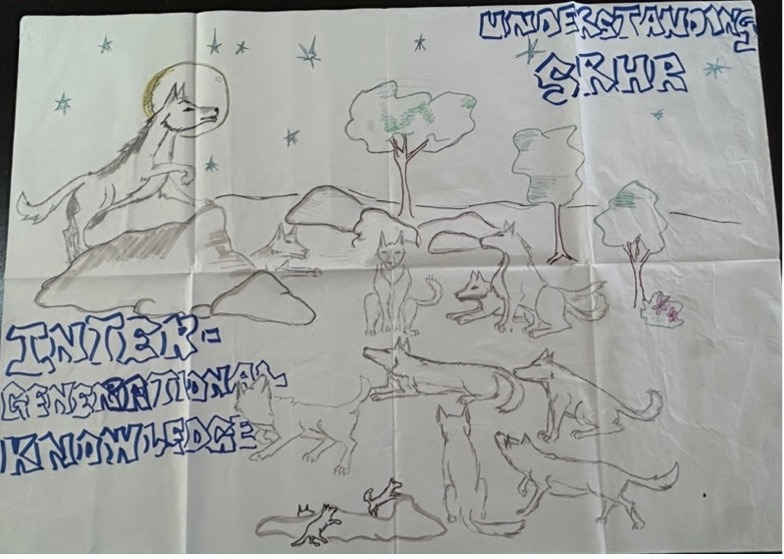
Illustration and narrative by a participant group. “So this wolf [represents] a leader, the turagi koro [chief], and these are the village community [who] look up to the king, the turagi koro, to follow his advice and messages…there [should] be more awareness programs, so that more [information] about SRHR rights [and] sex education can be given to youth at an early age [to help them know] what they can do in the future.”

As depicted by the illustration in figure 2, participants emphasised the significance of community hierarchies when it comes to gatekeeping of information. They advocated for increasing SRHR awareness within their communities so that village chiefs and elders could understand and enable youth to keep themselves safe.

Youth reported limited access to SRHR information within their communities and in school, with access controlled by gatekeepers such as community leaders and elders. This pushed them to seek inaccurate or harmful information online. The inability to access accurate SRHR information affected youth agency to understand and protect their SRHR, and increased risks of worse SRHR outcomes in disaster contexts.

### How contexts and spaces influenced agency before, during and after disasters

**Figure 3:**
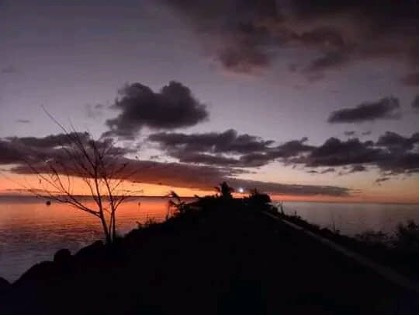
Photo of a sunset by Uci—*“*Most of them [youth] are not even aware of what SRHR is [and] they will be on their own [in disasters] …the light is the community and us young people we are like this darkness*.”*

Participants discussed how different contexts and power dynamics influenced their sense of belonging and inclusion, and their ability to access resources. Uci used a photo (figure 3) to explain that youth often felt like they were alone in the dark, without the knowledge or capability to protect their SRHR.

Kakala emphasised how social identities affected the sense of belonging for youth, explaining that sexual and gender diverse youth were not supported within his village. He expressed concern for younger youth that he could see were going through the same challenges.

‘For us LGBTQI, I have gone through a lot…we are not supported in the village… we can’t help it [being gay]. And in younger generation we can see them, they are following our footstep and they can’t help it.’ – Kakala

In disaster contexts, participants identified that displacement had a profound effect on their sense of safety and belonging. Drano explained that they were required to move to an evacuation hall in another village after every cyclone or volcano warning. She described how the lack of privacy in bathrooms and showers in the evacuation halls increased their risks of sexual harassment and abuse.

‘When we have a cyclone or a volcano warning …we have to move to another village. There’s a big hall [where] we have to stay until the warning has stopped… its unsafe for you to use washrooms and showers…They [boys and men] are all around and they watch’ – Drano

Dralakaka stated that sexual harassment and violence, and menstrual health were not viewed as priorities by village leaders. Consequently, these SRHR issues were not addressed in meetings, and those who experienced SRHR violations remained silent.

‘If it’s about sexual harassment, or menstruation, those types of issues they [village leaders] consider as a small issue. It has to be big issue for them to discuss it in meetings [so] when sexual harassment or domestic violence happens, the victims are usually silent’ – Dralakaka

Contexts and physical spaces such as villages and evacuation centres had profound impacts on youth agency. In non-disaster settings, youth already felt marginalised, and youth with sexual and gender-diversity faced further challenges to their sense of belonging and inclusion within their villages. When displaced from home in disaster contexts, the existing challenges increased SRHR risks such as violence, harassment and lack of privacy especially for menstrual health needs, compounded by a lack of focus on youth SRHR by decision-makers.

### How immediate priorities affected agency to protect SRHR in the aftermath of disasters

Youth SRHR were influenced by immediate needs and ability to access resources during disasters. Makosoi explained how basic human rights to access water, food, shelter and clothing were eroded after disasters.

‘You don’t have [basic] human rights…If the house is gone you don’t have shelter, food, clothes… you don’t have sanitary stuff, ways to take care of [menstrual health]… Rights are being washed away [after] the flood has already washed [away] your house’ – Makosoi

Access to clean water was a major challenge after disasters, which increased multiple SRHR risks. Senibua discussed how they travelled in groups to the river for menstrual health management, to minimise risks of sexual and gender-based violence.

‘During disaster when we have menses, there’s no water available. We go to the river and clean up there…we gather some friends and some of our relatives who go with [us], [to] protect ourselves [from sexual and gender-based violence]’ – Senibua

Participants reported that faced with limited resources, youth were forced to offer transactional sex to access basic needs. Dididi discussed how youth engaged in transactional sex in exchange for food or money for their families, thereby also increasing risks of early pregnancy.

‘When there’s not enough money for food … some girls tend to sell themselves just to gain money to feed the family, and ended up being pregnant.’ – Dididi

Similarly, Daunini explained that main income source for families in the maritime zones came from their daughters working in tourist hotels. When hotels were closed in the aftermath of disasters, some young women were pressured into transactional early marriage to support the family.

‘Parents allow [young women] to get married to an older guy, to get source of living…In islands they depend on their daughters working …In disasters, no hotel is open so somehow they have to survive’ – Daunini

Kakala explained how sexual and gender diverse youth felt unsafe going to evacuation centres. Consequently, they had to make choices between safe shelter and access to other resources such as food. Kakala reported that even if they found their own safe spaces, they still had to risk going to evacuation centres to access food through their families.

‘We don’t feel they can go to evacuation centre, would rather stick in a group, we will all live together in the same safe house…[but then] we go to our own family and get the food from the family for us to eat’ – Kakala

**Figure 4:**
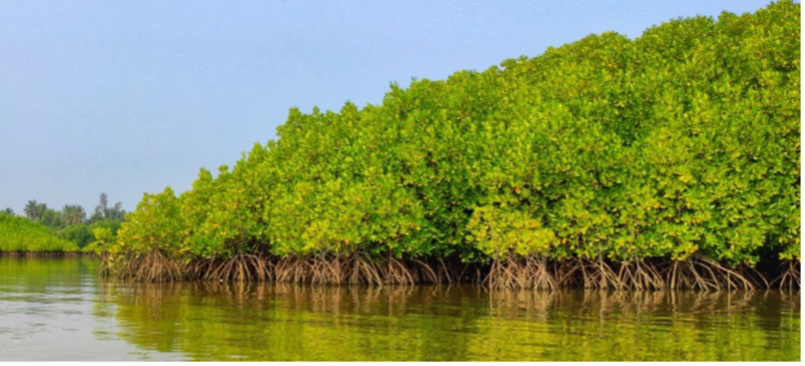
Photo of mangroves, image by Vishwasa Navada K on Unsplash. “We make use of available resources…during disasters, hospitals and health centres close down, so we use mangroves as herbal medicine…[and also] as food resources. [You can] get crabs from mangroves and use it for family income…it’s useful for the environment…we use a piece of cloth during menses …if there’s no deodorant [we] use lemon as a deodorant.” – Senibua.

In the context of limited SRHR information and services after disasters, participants highlighted the use of available resources from their environment to mitigate some of the challenges. Many participants described using cloth for menstrual hygiene when unable to access menstrual products, boiling water from the creeks and rivers when faced with water scarcity, and using lemons for personal hygiene. Senibua used a photo of mangroves (figure 4) to explain how mangroves protected the environment and provided a source of herbal medicine, food and income for their families.

For youth, limited access to basic resources such as shelter, food and water intersected with limited financial resources. Meeting basic needs often increased risks to their SRHR, as youth were having to engage in transactional early marriage or offer transactional sex for access to resources. Participants highlighted existing resources within their environment such as mangroves which helped meet some basic needs such as income or food, thereby mitigating some of the SRHR risks.

### How daily SRHR risks influenced youth perception of risk and limited agency before, during and after disasters

Participants discussed the daily risk environments in which they lived, which limited their SRHR agency. Irrespective of other hazards, their experiences in daily life influenced their perceptions of what risk was and what their rights were. Cevuga explained that violence was normalised, with many youth growing up in environments where they frequently experienced sexual, emotional and verbal abuse.

‘People consider it [sexual and gender-based violence and family violence] normal. Some grew up in a very toxic environment, where they have been sexually abused, emotionally abused, verbal abuse…so they consider it normal’ – Cevuga

The normalisation of violence extended beyond the home to their communities. Buasala explained that especially within religious communities, youth were not encouraged to seek support or speak about violence that they experienced or witnessed. She stated that the expectation was to suffer in silence and get through it.

‘The thing is we don’t talk about [violence] at home… especially if you’re in church it’s like oh you just have to go through it…eventually there’s light at the end of the tunnel…They don’t teach at home to come out and seek help or reach out to someone. [You’re expected to] suffer in silence…until you just make it out alive’ – Buasala

The conditions that they lived in often drove youth to leave home and survive on the streets, which then increased likelihood of having to engage in transactional sex to seek shelter and resources.

In rural areas, participants described how the pressure and expectation to provide for family increased risks of early marriage, with many young women leaving school early to do so. Dididi explained how these pressures increased during COVID-19 related lockdowns:

‘Especially in the rural areas, in my village there were lots of, during COVID-19 lockdown there were many young girls and young boys in year 9, year 10, they left school… some of them got married at a very early age – 16, 17 years’ – Dididi

With these examples, participants demonstrated that the SRHR risks they faced every day caused disaster conditions for youth even in the absence of natural hazards. Makosoi expressed how the mental and physical impacts of family violence, intimate partner violence and rape were perceived as disasters, especially for young girls.

‘When we look at a disaster it can be natural terms or even in family situations…[family violence, intimate partner violence] it’s a disaster that can cause you mental trauma…if a girl is raped, it’s a disaster for her’- Makosoi

**Figure 5:**
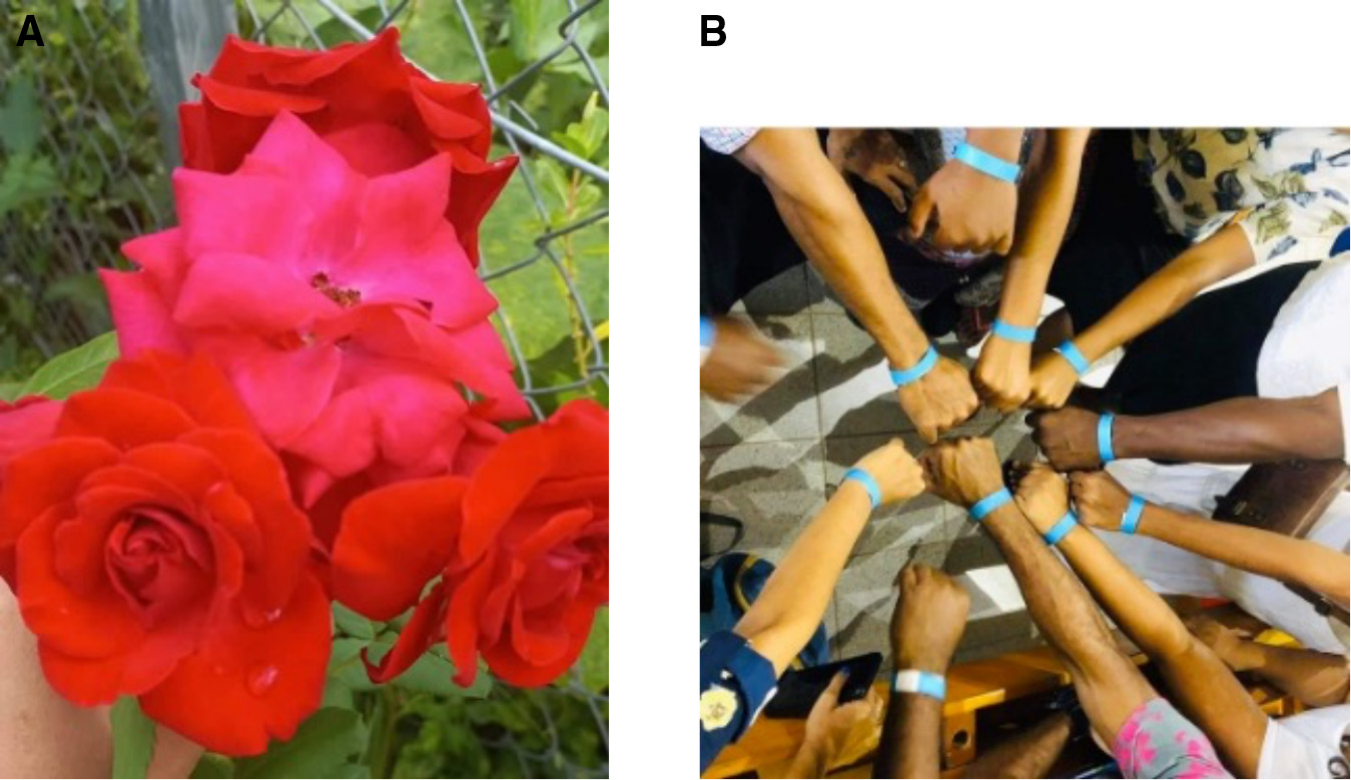
(A) Photo of roses by Makosoi—*“*Being healthy is more (than) having a healthy body, it is the right for every individual to have a safe environment to live in… If you planted a rose, it’s not like you leave it and it will bloom one day…in order to bloom you have to water the rose everyday”. (B) Photo title ‘We become resilient when we are united’ by Cevuga—”This picture signifies power and strength when young people are united. Disasters are inevitable, but resilience can only be possible when youth come together.”

Participants used photos to express their perceptions of everyday risks and disasters (figure 5). Makosoi used a photo of a rose (figure 5A) to explain the importance of a nurturing and healthy environment for enabling youth to protect themselves and fulfil their SRHR. Equating roses to youth, she described how roses could only bloom if they were watered daily and had access to an enabling environment that allowed them to thrive. Similarly, Cevuga used a photo (figure 5B) to highlight the importance of unity for empowerment and resilience. She stated that in the contexts that they lived in, disasters were inevitable, but that youth had power and strength when they worked together.

Participants identified daily SRHR risks that youth faced within the contexts that they lived in. Factors such as family pressure and violence within homes limited youth agency and influenced their perception of disasters and SRHR risks. Youth identified the importance of a nurturing environment and the power of unity in helping build mental and physical resilience in youth before, during and after disasters.

## Discussion

We explored the perspectives of Fijian youth about fulfilling their SRHR in the context of increasing climate impacts. Our study revealed how existing challenges that constrained youth SRHR agency in their daily lives, such as limited access to SRHR information and services, and normalisation of violence within communities, further increased their SRHR risks in disaster contexts. Participants also highlighted how cascading effects (including multiple hazards, food and water insecurity, and economic insecurity) exacerbated inequities and negatively impacted youth SRHR agency. Faced with existing and new challenges that increased SRHR risks in disasters, participants used social peer networks and resources such as mangroves to mitigate some of the risks.

**Figure 6:**
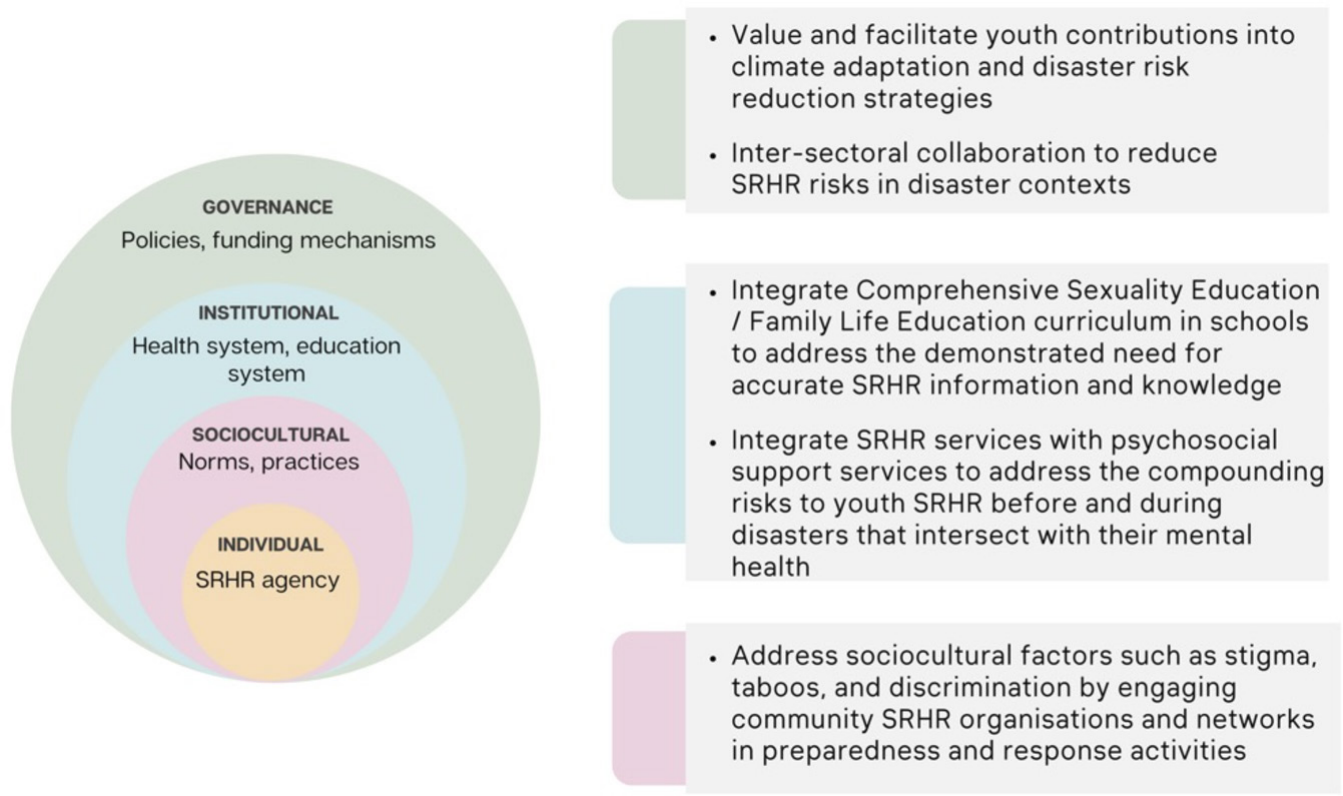
Summary of key implications for increasing individual sexual and reproductive health and rights (SRHR) agency through addressing factors across sociocultural, institutional and governance levels. The colours correspond to the relevant levels.

As a fundamental human right, agency is embedded within the sustainable development goals target for universal access to SRHR (Target 5.6).^16^ Participants highlighted multiple individual, community and structural factors that limited youth agency to protect their SRHR before, during and after disasters. The importance of intersectoral and multilevel approaches to address youth SRHR has been widely addressed and discussed in non-disaster literature.^23 39 40^ Our findings reinforce the importance of addressing existing challenges by demonstrating how challenges were exacerbated in disaster contexts. Findings corroborate perspectives from research with internally displaced youth in Haiti, highlighting how complex multilevel factors influence agency within humanitarian contexts.^41 42^ The authors’ suggestion that agency is a non-linear and incremental process likewise reflects the accounts of Pacific youth. We therefore discuss four key implications identified through our participatory process that have the potential to promote youth SRHR agency across multiple levels. Figure 6 summarises these key implications, which were designed to address the four themes identified by our research (figure 1), through interventions at sociocultural, institutional and governance levels.

First, we advocate for increasing access to youth SRHR information and knowledge, which is currently limited by community gatekeepers and variable quality of FLE in schools. Schools have the potential to improve youth SRHR through Comprehensive Sexuality Education (CSE).^43^ CSE is an age-appropriate and culturally relevant approach to teaching scientifically accurate, non-judgemental information about sexuality and relationships.^44^ The United Nations technical guidance on CSE developed by UN agencies and the WHO aims to provide young people with the knowledge, attitudes and skills to protect and advocate for their health, well-being and dignity.^44^ Recognising that other names for CSE such as ‘life skills’, ‘holistic sexuality education’, or ‘family life’ may reflect differences in emphasis of the education programme, the guidelines provide a unified approach for empowering young people to understand and protect their rights. Globally, CSE is associated with improved SRHR outcomes such as reduced early and unintended pregnancies, and better overall youth health and well-being.^45^ Despite the benefits of CSE, broader contextual factors related to cultural and religious beliefs, the capability and willingness of teachers to teach it, and limited global and national commitments continue to affect the extent to which CSEs are implemented and integrated globally.^46^ The fact that CSE is called FLE in Fiji suggests that SRHR stigma influences how sexuality education is perceived and discussed. A recent global survey on the status of CSE noted that while Fiji referred to the availability of a sexuality curriculum, the level of education or the topics covered were not specified.^47^ Our findings highlighted that in the absence of accessible information youth turned to harmful resources such as online pornography for SRHR information. A unified approach to CSE in Fijian school curriculums is important to address the demonstrated need for accurate and respectful SRHR information and knowledge. An enabling environment for CSE also requires mitigating community misconceptions and investing in training and support for teachers to deliver accurate, non-judgmental SRHR information. Facilitating youth access to comprehensive SRHR information that is situated within their cultural contexts is a key component of increasing their SRHR agency.

Second, we advocate for addressing sociocultural factors that increased participant SRHR risks and limited their agency. Factors such as normalisation of sexual and gender-based violence and family violence created disaster conditions for youth. In the aftermath of disasters, participants described increased violence and additional risks such as transactional early marriage. Stigma and discrimination caused sexual and gender diverse youth to feel unsafe in their communities and homes, and further increased risks to their safety due to violence towards them in evacuation centres. Further, exclusion of youth from decision-making spaces, and SRHR issues not being prioritised by community leaders, pushed those who experienced violations during disasters into silence. We have previously reported how community hierarchies increased youth SRHR risks in the disaster responses to the 2020 Tropical Cyclone Harold in Fiji, Vanuatu and Tonga.^31^ Findings from this study reiterate how the exclusion of youth from decision-making spaces intersect with factors such as stigma and discrimination to compound youth SRHR risks. Pacific community organisations have demonstrated strengths in how they use social capital to engage with community leaders and youth members to address SRHR stigma and navigate taboos.^33^ The knowledge of context and existing relationships with community members and leaders were key to how community organisations increased youth SRHR service provision and access in disaster contexts.^33^ This was especially crucial for youth with sexual and gender diversity. Our findings support the need for engaging Pacific community SRHR organisations and networks to integrate context-specific youth SRHR service provision and access in climate adaptation and disaster risk reduction strategies.

Third, we advocate for facilitating youth contributions into national and regional climate and disaster policies and strategies to reduce future risks. Facilitating meaningful youth engagement is a global priority in disaster risk reduction and sustainable development agendas. However, youth continue to face structural barriers such as ageism, societal hierarchies and limited political will and funding.^48^ Using examples of how youth supported each other to mitigate risks of sexual and gender based violence, participants demonstrated youth leadership and strong social capital. Peer support networks were also highlighted as essential for strengthening their resilience to withstand SRHR risks before, during and after disasters. Participants showed resourcefulness in how they utilised natural resources such as mangroves to reduce risks related to financial and food insecurity. The creativity and innovation of youth were also evident from their engagement with the research process, using diagrams and photographs to discuss their concerns. Our findings support an increasing body of evidence highlighting how youth contribute creative solutions for addressing disaster risks.^49 50^

Finally, the intersecting and compounding factors that affected youth agency offer opportunities to strengthen intersectoral collaborations to reduce SRHR risks. Collaborations across health, WASH and shelter sectors are crucial for reducing risks of sexual and gender-based violence in evacuation centres and WASH facilities. Importantly, findings demonstrate how economic, food and water insecurities all intersected and exacerbated risks to youth SRHR. The concurrence of water and food insecurities due to climate impacts is known to exacerbate each other and have adverse impacts on mental and physical health.^51^ Our findings suggest that with the background of daily SRHR risks that affect youth mental and physical health, the additional SRHR risks of compounding economic, food and water insecurities may considerably worsen their mental health. Findings highlight the crucial need to integrate SRHR services with psychosocial support services for youth.

To our knowledge, this is the first study exploring how Fijian youth perceive climate impacts and disaster risks related to their SRHR. We provide important insights into the multiple factors that limited youth agency, and how their perceptions of SRHR and disaster risks were influenced by the multihazard contexts and daily SRHR risks they experienced. By using participatory and visual methods, we demonstrate how empowering youth with the choices for creative expression of their views provided a holistic understanding of youth SRHR risks. While the research was limited to youth from the Western district of Fiji, findings contribute crucial insights into the links between youth SRHR risks and the increasing impacts of the climate crisis.

## Conclusions

Our study shows the urgent need for an intersectional and whole-of-society approach to promote SRHR agency and reduce climate impacts on the SRHR of Pacific youth. We underscore the importance of addressing existing social and health inequities which exacerbated risks for youth SRHR in disaster contexts. Reinforcing the synergy between climate change adaptation and disaster risk reduction, safeguarding youth SRHR requires understanding existing SRHR risks, and strengthening climate and disaster governance to address these risks. As the largest population in the region, youth are key to building resilience and advancing sustainable development in the Pacific. Ensuring equitable and fair responses to the climate crisis requires identifying and addressing injustices that disproportionately increase SRHR risks for Fijian youth.

## Data Availability

Data are available upon reasonable request. Data included in the manuscript are participant-provided, anonymised data including photo narratives, illustrations and qualitative interview transcripts exploring their lived experiences. Pseudonyms (Fijian flower names) were used for all participants.
