# Supplementary material for: Changing climates, compounding challenges: a participatory study on how disasters affect the sexual and reproductive health and rights of young people in Fiji
Source: BMJ Glob Health. 2023 Dec 15;8(Suppl 3):e013299. doi: 10.1136/bmjgh-2023-013299 (PMC10729163; doi:10.1136/bmjgh-2023-013299)
Supplement: Supplementary data [file bmjgh-2023-013299supp001.pdf]

Standards for Reporting Qualitative Research (SRQR)*

http://www.equator-network.org/reporting-guidelines/srqr/

Title and abstract

	Page/section(s)
**Title** - Concise description of the nature and topic of the study Identifying the study as qualitative or indicating the approach (e.g., ethnography, grounded theory) or data collection methods (e.g., interview, focus group) is recommended	Title page
**Abstract** - Summary of key elements of the study using the abstract format of the intended publication; typically includes background, purpose, methods, results, and conclusions	Title page

Introduction

**Problem formulation** - Description and significance of the problem/phenomenon studied; review of relevant theory and empirical work; problem statement	Section 1 Introduction
**Purpose or research questio**n - Purpose of the study and specific objectives or questions	Last paragraph of introduction

Methods

**Qualitative approach and research paradigm** - Qualitative approach (e.g., ethnography, grounded theory, case study, phenomenology, narrative research) and guiding theory if appropriate; identifying the research paradigm (e.g., postpositivist, constructivist/ interpretivist) is also recommended; rationale	Section 2, Methods, theoretical approach (section 2.1)
**Researcher characteristics and reflexivity** - Researchers' characteristics that may influence the research, including personal attributes, qualifications/experience, relationship with participants, assumptions, and/or presuppositions; potential or actual interaction between researchers' characteristics and the research questions, approach, methods, results, and/or transferability	Appendix A (reflexivity statement) and section 2.6 - Ethics and researcher reflexivity
**Context** - Setting/site and salient contextual factors; rationale	Introduction section 1.1, Methods, Study setting (section 2.2)
**Sampling strategy** - How and why research participants, documents, or events were selected; criteria for deciding when no further sampling was necessary (e.g., sampling saturation); rationale	Methods section, Participants and recruitment (section 2.3)
**Ethical issues pertaining to human subjects** - Documentation of approval by an appropriate ethics review board and participant consent, or explanation for lack thereof; other confidentiality and data security issues	Methods, Ethics and researcher reflexivity (section 2.6)

Murphy N, *et al. BMJ Glob Health* 2023; 8:e013299. doi: 10.1136/bmjgh-2023-013299

**Data collection methods** - Types of data collected; details of data collection procedures including (as appropriate) start and stop dates of data collection and analysis, iterative process, triangulation of sources/methods, and modification of procedures in response to evolving study findings; rationale	Methods, Data collection and management (section 2.3)
**Data collection instruments and technologies** - Description of instruments (e.g., interview guides, questionnaires) and devices (e.g., audio recorders) used for data collection; if/how the instrument(s) changed over the course of the study	Methods, Data collection and management (section 2.4)
**Units of study** - Number and relevant characteristics of participants, documents, or events included in the study; level of participation (could be reported in results)	Findings (section 3), Table 1 .
**Data processing** - Methods for processing data prior to and during analysis, including transcription, data entry, data management and security, verification of data integrity, data coding, and anonymization/de-identification of excerpts	Methods, Data analysis (section 2.5)
**Data analysis** - Process by which inferences, themes, etc., were identified and developed, including the researchers involved in data analysis; usually references a specific paradigm or approach; rationale	Methods, Data analysis (section 2.5)
**Techniques to enhance trustworthiness** - Techniques to enhance trustworthiness and credibility of data analysis (e.g., member checking, audit trail, triangulation); rationale	Methods, Data analysis (section 2.5)<br>Ethics and research reflexivity (section 2.6)

Results/findings

**Synthesis and interpretation** - Main findings (e.g., interpretations, inferences, and themes); might include development of a theory or model, or integration with prior research or theory	Findings (section 3)
**Links to empirical data** - Evidence (e.g., quotes, field notes, text excerpts, photographs) to substantiate analytic findings	Findings (section 3)

Discussion

**Integration with prior work, implications, transferability, and contribution(s) to the field -** Short summary of main findings; explanation of how findings and conclusions connect to, support, elaborate on, or challenge conclusions of earlier scholarship; discussion of scope of application/generalizability; identification of unique contribution(s) to scholarship in a discipline or field	Discussion (section 4) including key implications and Figure 6
**Limitations** - Trustworthiness and limitations of findings	Final paragraph of Discussion (section 4)

Other

**Conflicts of interest** - Potential sources of influence or perceived influence on study conduct and conclusions; how these were managed	Last page<br>- Author contributions, funding

**Funding** - Sources of funding and other support; role of funders in data collection, interpretation, and reporting	Last page - Funding

*The aims of the SRQR are to provide transparency in all aspects of qualitative research by providing guidelines for reporting qualitative research

Reference:

O'Brien BC, Harris IB, Beckman TJ, Reed DA, Cook DA. **Standards for reporting qualitative research: a synthesis of recommendations.** *Academic Medicine*, Vol. 89, No. 9 / Sept 2014 DOI: 10.1097/ACM.0000000000000388
