# Supplementary material for: Changing climates, compounding challenges: a participatory study on how disasters affect the sexual and reproductive health and rights of young people in Fiji
Source: BMJ Glob Health. 2023 Dec 15;8(Suppl 3):e013299. doi: 10.1136/bmjgh-2023-013299 (PMC10729163; doi:10.1136/bmjgh-2023-013299)
Supplement: Supplementary data [file bmjgh-2023-013299supp002.pdf]

Appendix A – Reflexivity Statement

1. How does the study address local research and policy priorities?

The overall doctoral research study was envisaged by NM when she was working in an international non-governmental organisation for SRHR and collaborating with Pacific Member Associations while based in Australia. This participatory research component was designed in collaboration with the Fijian Youth Sexual and Reproductive Health and Rights Alliance (FYSA) in response to an identified need. FYSA is a youth network with a focus on empowering Fijian youth in all their diversity and facilitating youth-driven solutions to advance youth SRHR. One of the outputs from the research collaboration will be a report that FYSA can disseminate with their networks and partners in the region, to influence policy and support local, regional, and global advocacy.

2. How were local researchers involved in study design?

The project was designed in collaboration and partnership with TR from the Fijian Youth Sexual and reproductive health and right Alliance (FYSA). NM and TR were co-researchers through all aspects of the participatory project, including planning and conducting workshops, interviewing youth participants, analysing data, and reporting findings. Two youth members of FYSA (AA and IK) assisted and contributed to both workshops and in analysing data. A local SRHR and mental health counsellor (KB) from a Pacific SRHR organisation delivered a mental health preparedness session and was available for SRHR counselling for participants throughout and after the project.

3. How has funding been used to support the local research team?

Funding was used to pay the FYSA research team (TR, AA and IK) and KB for their time, expertise, and contributions to the project. This included valuing their time in planning the project, conducting the research activities, and their contributions to the reporting process. We also used funding to reimburse all participants for contributing their experiences and perspectives to the study. We provided food and refreshments for all workshops, mobile data money for individual photographs, narratives, or illustrations, and reimbursed transport costs for attending the workshops and individual interviews.

4. How are research staff who conducted data collection acknowledged?

This paper is one of several papers from the overall research study. All members of the Fiji research team have contributed as co-authors on this paper and are co-authors on other papers from the participatory project.

5. Do all members of the research partnership have access to study data?

All data was de-identified by NM and securely stored in a OneDrive folder through NM's University of Melbourne account. All members of the research partnership have access to the de-identified transcripts and de-identified participant-derived data including photographs, narratives, and illustrations from the participatory project.

6. How was data used to develop analytical skills within the partnership?

Data analysis was a collaborative process between NM, TR, AA and IK. Over a period of 1.5 weeks we reflected on the data and collectively generated all codes and themes through a reflexive analysis process. We designed posters of the themes, using de-identified illustrative quotes and participant-derived visual data, sharing these with the participants to invite reflection and further dialogue. Aligned to participatory research, this was designed to enable youth participants to engage in the analysis process, contributing and developing their analytical skills as part of the research process. Further, the research team are co-authors in reporting findings in academic journals.

7. How have research partners collaborated in interpreting study data?

As mentioned in points #3 and #6, all members of the research team were involved in data analysis and reporting. As the overall research approach intends to identify opportunities for transformative change, we analysed the data aiming to understand and discuss the implications for policy and practice.

8. How were research partners supported to develop writing skills?

The research team included senior academics (KJB, PA and MAB) who provided guidance and supervision for NM's PhD research. NM is a PhD researcher who has experience in writing for policy advocacy and reporting project outcomes to donors. The Fiji research team of TR, AA, IK and KB have combined experience in writing for grassroots advocacy and reporting to donors. Throughout the research process, we developed our academic writing skills guided by the senior academic supervisors.

9. How will research products be shared to address local needs?

All papers resulting from this study will be published as open access and disseminated at relevant conferences as oral or poster presentations. We are summarising key findings as infographics to share with all participants and interested stakeholders in the Pacific. Further, NM and FYSA team are collaborating to summarise findings in a report that can be owned by FYSA, for sharing widely with their networks and partners.

10. How is the leadership, contributing, and ownership of this work by LMIC researchers recognised within the authorship?

As mentioned in point #4 all members of the research team are co-authors. As NM and TR led the participatory project, they are first and second authors respectively. As the study is part of NM's overall research project, NM is the corresponding author on all papers from the research.

11. How have early career researchers across the partnership been included within the authorship team?

The first and corresponding author (NM) is a PhD researcher and the study is part of her overall PhD research. The Fiji research team are community practitioners who are interested in developing skills in research and academic writing, to bridge the gap between community advocacy and academic publications in research.

12. How has gender balance been addressed within the authorship?

The research team is diverse in gender, race, nationalities, and sexual orientations. To respect researcher privacy, we are choosing not to be too specific, but the authors include five women, two men, and one non-binary author.

13. How has the project contributed to training of LMIC researchers?

The overall study has contributed to NM's PhD research, strengthening her knowledge, skills and research training. Reflecting our participatory and collaborative research approach, the project increased knowledge and skills of the research team in relation to academic research practices and requirements. Further, by inviting participants to reflect on findings and engage in further dialogue based on their reflections, we contributed to enhancing knowledge and research analysis skills in participants.

14. How has the project contributed to improvements in local infrastructure?

This project had no direct contribution to improvements in local infrastructure.

15. What safeguarding procedures were used to protect local study participants and researchers?

As SRHR issues are sensitive and taboo subjects in most Pacific contexts, we developed a risk prevention and mitigation strategy that guided our study design to ensure participant safety was a priority. For instance, the first session of the first workshop was facilitated by KB, who is a trained SRHR and mental health counsellor. She guided participants in understanding how to recognise, identify, and manage emotions, and we discussed when to ask for support and how to access support. KB was available throughout the project and engaged confidentially with any participants who needed to talk through anything that came up for them. Participants were also able to contact any member of the research team for further discussion or support. The research team of NM, TR, AA, IK and KB engaged in regular debriefing and reflexive discussions to ensure we were identifying and managing our own emotions throughout the project.

Murphy N, *et al. BMJ Glob Health* 2023; 8:e013299. doi: 10.1136/bmjgh-2023-013299
